# Framing to reduce present bias in infrastructure design intentions

**DOI:** 10.1016/j.isci.2022.103954

**Published:** 2022-02-19

**Authors:** Patrick I. Hancock, Leidy Klotz, Tripp Shealy, Eric J. Johnson, Elke U. Weber, Katelyn Stenger, Richa Vuppuluri

**Affiliations:** 1 Department of Engineering Systems and Environment, University of Virginia, 114 Olsson Hall 151 Engineers Way, P.O. Box 400747, Charlottesville, VA 22904, USA; 2 Columbia Business School, 3022 Broadway, New York, NY 10027, USA; 3 Charles Edward Via, Jr. Department of Civil and Environmental Engineering, Virginia Tech, 200 Patton Hall, Blacksburg, VA 24061, USA; 4 Department of Psychology, Princeton University, 309 Peretsman Scully Hall, Princeton, NJ 08540, USA

**Keywords:** Civil engineering, Construction engineering, Interdisciplinary application studies, Sociology

## Abstract

Infrastructure professionals (N = 261) were randomly assigned to either a future or present-framed project description and asked to recommend design attributes for an infrastructure project. The future-framed condition led professionals to propose a significantly longer infrastructure design life, useful life to the community, and acceptable return on financial investment. The findings suggest a straightforward and inexpensive way to lessen present bias in various design contexts

## Highlights

• Designers were temporally primed prior to engaging in a design decision scenario
• The prime was delivered through word tense changes to a request for proposal
• The future orientation significantly improved the sustainability of design concepts
• Results indicate present bias can be reduced through changes to design documents

## Graphical Abstract

**Figure:**
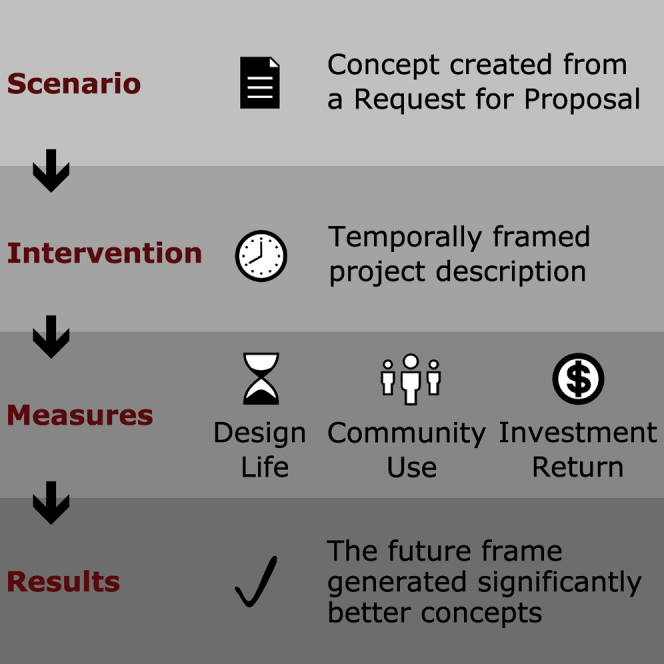

## Introduction

Infrastructure systems provide essential water, shelter, mobility, and other services. There is a global need to update these systems and to expand and adapt them to serve the billions who do not currently have access (Department of Economic and Social Affairs, 2015). At the same time, existing infrastructure accounts for around one-fifth of climate changing emissions (Creutzig et al., 2016), and new infrastructure development that is more of the same would independently exceed the carbon budget needed to avoid the worst effects of climate change (Müller et al., 2013). Present-day choices about infrastructure will impact how effectively the water, shelter, and mobility needs of current and future generations are met, and present-day decisions about infrastructure will impact whether we maintain a set of safe conditions for life on earth.

Decisions made by a variety of project stakeholders long before construction begins play an outsized role in determining the costs and benefits of an infrastructure project (Sen, 2001). Previous work has discovered that, as in other domains (Johnson et al., 2012), the context in which such decisions about infrastructure are made can impact the outcome (Harris et al., 2016; Shealy et al., 2016, 2018). For example, compared to a control group, professional infrastructure engineers made aware of high achieving "role-model" projects set 34% more ambitious goals for sustainability (Harris et al., 2016). Consequently, one important path to more sustainable infrastructure is to identify the specific decision contexts that motivate designers (e.g., engineers, urban planners, and architects) to create more sustainable outcomes.

People’s construal of time can lead to more or less sustainable outcomes (Trope and Liberman, 2010). One barrier to more sustainable choices is present bias: individuals' tendency to irrationally prefer options with more immediate benefits over options with delayed benefits. Present bias can be overcome by priming future considerations so that they are considered first or more extensively. One way in which this is done is through structured use of construal level theory (CLT), which describes the malleability of psychological distance, to alter an individual’s construal of the decision context (Pronin et al., 2008; Trope and Liberman, 2010; Weber et al., 2007). For example, close psychological distance to the effects of climate change is correlated with increased concern for climate change impacts (Maiella et al., 2020; Spence et al., 2012). Similarly, a closer perceived distance to the future might elicit long-term sustainable outcomes by reducing present bias.

In CLT, temporal orientation refers to how people perceive and value time, in terms of past, present, or future. Research has shown that eliciting a future orientation can lessen present bias in a variety of decision contexts. For example, having people contemplate future outcomes before current alternatives can lessen present bias in a variety of decision contexts (e.g. organ donation (Johnson and Goldstein, 2003) and retirement planning (Earl et al., 2015; Thaler and Shlomo, 2004)) and increase patience more generally (Weber and Johnson, 2015). This research explores whether eliciting a future orientation toward a design task, to lessen present bias, generates more sustainable concepts for infrastructure design.

In practice, infrastructure design often begins with a request for proposals, which is a document outlining the initial requirements for design firms that wish to bid on a project. It communicates to designers the project intent of whoever is paying, often a government or municipality on behalf of taxpayers. The request for proposals is therefore an influential avenue for CLT interventions that might make individual designers more likely to generate sustainable design concepts during the preliminary phases of an infrastructure design project.

Here, we examined whether a future orientation, delivered via an online survey and introduced via randomly assigned changes to the word tense in a request for proposals document (see STAR methods), would elicit professional infrastructure designers to take a longer-term view in their preliminary design concepts for an infrastructure project. The study population was comprised of certified Envision professionals. Envision is a sustainable design framework supported by the Institute of Sustainable Infrastructure. The Envision population was chosen because it is comprised of professionals with experience in infrastructure design who, by earning Envision certification, have demonstrated a commitment to enhancing infrastructure sustainability. Because our intervention targets the construal level, we expect, but cannot be sure, that results from this group would extend to professionals lacking a similar commitment to sustainability.

## Results

**Figure 1:**
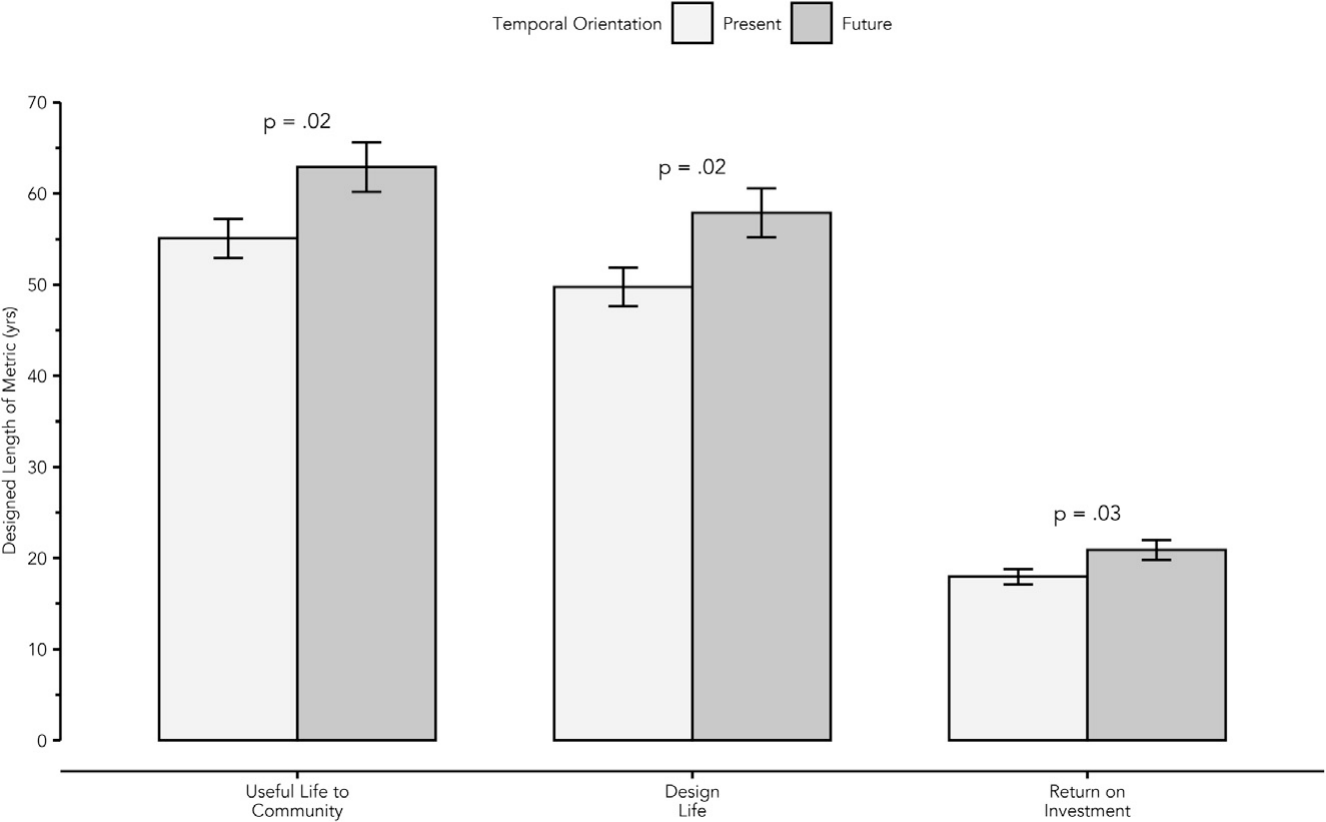
A future orientation led to a statistically significant increase in useful life to community, design life, and return on investment. Error bars represent ±1 SE.

The experimental deployment was able to collect N = 261 participants with n = 147 in the present group and n = 114 in the future group. While the group assignment was random and evenly assigned, the future group had a higher dropout rate than the present group for reasons that are unknown. The data were then analyzed across the participants' responses for the design task via the three dependent variables of design life, useful life to the community, and maximum acceptable return on investment. For each dependent variable, the null hypothesis was that no significant difference exists between participants in the future-framed or present-framed group. A detailed breakdown of the statistics can be found in Table 1. Visual display of the main findings is in Figure 1. Findings related to each hypothesis are as follows:

**Table 1:** Data analysis results

Variable	Sample	Mean (SD)	95% CI	t	df	p	Cohen’s d	Pearson’s r
Design life			[1.41, 14.83]	2.26	229.09	.02	0.31	0.16
Present	147	49.75 (25.58)						
Future	114	57.88 (28.53)						
Community			[0.99, 14.62]	2.39	228.96	.02	0.30	0.15
Present	147	55.09 (28.99)						
Future	114	62.89 (26.01)						
ROI			[0.24, 5.63]	2.14	227.67	.03	0.28	0.14
Present	147	17.95 (10.23)						
Future	114	20.89 (11.51)						

Presents the statistical results of the three t tests of dependent variables across the present and future groups.

**Table 2:** Descriptions of the credits from the Envision Framework used within the study

Envision Credit	Description
Quality of Life 3.1	Advance Equity & Social Justice
Leadership 1.3	Provide for Stakeholder Involvement
Leadership 2.2	Plan for Sustainable Communities
Leadership 2.3	Plan for Long-Term Monitoring & Maintenance
Leadership 2.4	Plan for End-of-Life
Leadership 3.3	Conduct a Life Cycle Economic Evaluation
Resource Allocation 2.1	Reduce Operational Energy Consumption
Resource Allocation 3.2	Reduce Operational Water Consumption
Climate and Resilience 1.2	Reduce Greenhouse Gas Emissions
Climate and Resilience 2.1	Avoid Unsuitable Development

The participants who received the future-oriented request for proposal set a significantly longer targeted useful life to the community (t = 2.26, df = 229.09, MD = 7.80, p = 0.02) compared to those in the present group. Useful life to the community is an essential measure of a comprehensive sustainable infrastructure design (Valdes-Vasquez and Klotz, 2013). This measure influences how limited available capital benefits society now and into future (Sierra et al., 2017). All else being equal, a longer useful life is more sustainable.

The participants who received the future-oriented request for proposal also construed a significantly longer design life (t = 2.39, df = 228.96, MD = 8.12, p = 0.02) compared to those in the present group. Targeting a longer design life obligates designers to mitigate a wider array of uncertain future risks, such as climate change, through their design decisions (Hallegatte, 2009). Increases to the design life of roadway infrastructure, for example, have been shown to contribute to improvements in the sustainability of the project’s life cycle (Al-Qadi et al., 2015).

Finally, the participants in the future-orientated group were willing to accept a significantly higher number of years for the return on investment (t = 2.14, df = 227.67, MD = 2.93, p = 0.03) than those in the present group. This suggests that the future framing might be one way to mitigate time-inconsistency in designer decision-making (e.g., present bias—where small payoffs now are preferred over larger payoffs in the future) to allow for sustainable infrastructure across a wider temporal frame (Henderson and Bateman, 1995).

While our findings suggest a way to lessen present bias, they did not confirm that doing so would translate to higher levels of achievement through the Envision rating system (see Table 2 for the credits used in this study). No significant differences in Envision credits were found from the participant’s temporal orientation. Perhaps, participants found the Envision credits used unrelated to the temporal construal of the project. Or perhaps the participant’s future orientation had “worn off” by the time they engaged with Envision credits later in the study. Future research could examine whether stronger future framing (i.e., through pictures or immersive virtual reality) directly introduced into Envision credits could lead to significant differences in sustainable achievement. Even absent other data, this research suggests that priming designers to have future orientation could elicit individual designers to generate more sustainable infrastructure design concepts, thus potentially opening the door for more sustainable design outcomes.

## Discussion

The future orientation, elicited through changes to word tense in a request for proposal, generated a longer-term perspective among designers who make decisions about sustainable infrastructure. Not all requests for proposals are present framed, but many are (e.g., the Gordie Howe International Bridge that connects Detroit, Michigan and Windsor, Ontario (Windsor-Detroit Bridge Authority, 2016), the Kosciuszko Bridge in New York City (New York State Department of Transportation), and the Los Angeles World Airports Automated People Mover (“Los Angeles World Airports. Request for Proposals for the Automated People Mover Landside Access Modernization Program at Los Angeles International Airport. 28 July 2017. BidNet. https://www.bidnet.com/bneattachments?/466507980.pdf)). Considering that major requests for proposals are often written in the present tense, greater attention to temporal framing in such requests could help designers ensure that the design concepts they come up with are more sustainable. Accordingly, priming a future orientation could provide a relatively straightforward and inexpensive way to reduce present bias.

These findings add to the growing evidence that construal-level interventions can elicit differences in designer decision-making. These research findings are relevant for practice in infrastructure and beyond. For example:

• legislators could require the use of future framing for infrastructure design projects;
• public agencies that issue request for proposals could modify their templates to be future oriented;
• future orientation could be strengthened in the wording of sustainability and climate action plans which are documents that, like requests for proposals, have long-term consequences; and
• future orientation could be strengthened with more explicit changes than word tense, such as explicit mentions of the future, visioning exercises (Dassen et al., 2016; Stein et al., 2016) and images of projected futures (Biliciler et al., 2021; Hershfield et al., 2011).

While this work suggests practical changes, the data only allow for limited speculation on the underlying psychological mechanisms driving the significant difference between the experimental groups. Future research could therefore examine whether heightened positive or negative emotion pathways mediate the observed effect (Van Boven and Ashworth, 2007), and whether episodic future thinking would enhance the presented effect, demonstrable, perhaps, via heightened prefrontal-mediotemporal interactions (Benoit et al., 2011). A deeper understanding of the underlying psychology could allow for more effective interventions and provide insights for how to apply these findings in other contexts.

### Limitations of the study

It is important to note a few limitations of the findings presented here. First, while significant results were found for the three dependent variables, the CIs for them are large. This suggests that the sample for this study was undersized. An effect size of d = 0.30 was observed. This was smaller than the expected effect size of 0.5 (see Star methods for more details). As such, extensions of this research could use studies powered to measure effect sizes that range from d = 0.2–0.5, thus allowing for the study of manipulation strength as well as less effective interventions. A larger sample population would be required to accomplish this.

Second, the study population of Envision professionals may already have a relatively strong future orientation; they have made a commitment to more sustainable infrastructure by joining the organization. Since the request for proposal interventions significantly impacted the decisions of this uniquely motivated group, we expect, but are not certain, that the interventions would similarly impact the decisions of designers who are less likely to be thinking of sustainability from the outset.

## STAR★Methods

### Key resources table

**Table:** 

REAGENT or RESOURCE	SOURCE	IDENTIFIER
**Deposited data**		

Envision User Participant Data	the Institute of Sustainable Infrastructure	

**Software and algorithms**		

R Project for Statistical Computing	https://www.rstudio.com	RRID:SCR_001905

**Other**		

Temporal Orientation Code	Zenodo	https://doi.org/10.5281/zenodo.5851386

### Resource availability

#### Lead contact

For further information and requests for resources please contact Patrick I. Hancock (pih9nc@virginia.edu).

#### Materials availability

This study did not generate any new materials.

### Experimental model and subject details

#### Human

Six-hundred and seventy-nine Infrastructure Design Professionals participated in this study. The participants recruited for this study were all Certified Envision Professionals who have experience in and are motivated to create sustainable infrastructure.

Written informed consent was obtained from each participant in accordance with the University of Virginia Institutional Review Board. Participants were excluded they failed any of the attention checks or left unanswered any of the questions pertaining to the studies primary DV’s. This resulted in a usable sample size of two-hundred and sixty-one participants.

### Method details

The study took the form of an online experimental decision scenario deployed through the Institute for Sustainable Infrastructure Envision professionals' email listserv. This amounted to a total population of 5,872 individuals. For completion of the experiment, participants received one credit hour towards the Envision certification continuing education requirement.

Professional infrastructure designers with training and experience with the Envision rating system served as the study population, due to their interest and expertise in creating more sustainable infrastructure. The Envision rating system is the leading sustainability rating system for infrastructure in the United States and is managed by the Institute for Sustainable Infrastructure.

Focusing on this specific set of designers served two purposes. First, if the intervention is successful it shows that sustainability focused behavioral interventions can elicit decision-making improvements for designers already committed to sustainably. Second, by indirectly generating improved sustainable decision-making, by priming the future, the impact of the intervention might be generalizable to non-Envision engineers, because a belief in sustainable actions might not be required for the intervention to be effective. In other words, these findings would add further evidence to present needs being privileged, in the minds of designers, over future needs in a way that hinders improved sustainable decision-making.

A two-group experimental design was created for this study. Since, to the knowledge of the authors, this is the first study that explores the impact of temporal orientation on designer decision-making, estimations were used to select the experimental parameters. The resulting experiment was designed to measure a medium effect size, d = 0.5, at an alpha of 0.05 with a power of 80%. As such a sample size of N = 250 was targeted, or n = 125 for each of the present and future groups.

The experimental manipulation was delivered via modified request for proposal document outlining a fictional infrastructure design decision scenario. This scenario casted participants as the lead engineer in charge of decision-making regarding a Water System Master Plan for the District of Sparwood in British Columbia, Canada (Sparwood, 2017). According, participants were tasked with answering questions regarding the design decisions they would make if they were to be tasked with completing the project. The experimental manipulation sought to prime participants with either a future or present orientation while they completed the design questions pertaining to the decision scenario.

#### Background information

The District’s vision statement introduced the participants the design decision scenario. Participants were instructed that the District of Sparwood is looking to provide and manage infrastructure and services—including potable water, sewage, storm water, and roads—cost-effectively and sustainably. The RFP explained to participants how the Water System Integrated Master Plan fit with the broader District vision. As such, the Master Plan focuses on the District's need for water distribution, wastewater treatment and collection, and storm water conveyance systems.

The participants were told that the creation of Water System Master Plan served "to provide strategic direction, support asset management initiatives, and assist the District in short- and long-term decision making". This includes financial, operational, and strategic considerations for how and when decisions should be made according to the plan. Participants were assigned the role of lead engineer for the District of Sparwood. Accordingly, this meant that the participant would oversee the creation of the Water System Master Plan. Additionally, as the lead engineer, their decision-making responsibility was to do what was best for the District by ensuring the project benefits outweigh its costs.

#### Comprehension checks

At this point, the participants were primed on the Envision framework requirements and the wastewater master plan project. The primes were broken up into multiple components to ensure prime strength and comprehension checks were used to ensure participants' understanding. For example, each participant was presented with a comprehension checks, form of a multiple-choice questions, after the project master plan details and the Envision written response instructions. By ensuring the manipulation was primed in the participants as intended, we would have confidence the results were pertinent to the questions we are looking to ask.

A total of 679 participants opened the decision scenario and consented to the experiment. Only a small portion of these participants completed the decision scenario. If the participants failed any of the comprehension checks or did not answer the three design characteristic questions, they were excluded from the study. Accordingly, we excluded 418 participants from the study analysis for a total sample size of 261 and the completion rate was 38%. As the study population were working professionals, the long completion time, of around 45-min, likely resulted in a large number of the dropouts.

#### Experimental manipulation

The participants received a manipulation in the form of the RFP project description. As mentioned above, the participants read information detailing Sparwood as it is now or what Sparwood envisions for the future. The descriptions only varied in temporal framing across the two experimental groups.

Below, the manipulation is listed in full. The future statement appears in brackets, and the present statement appears in parentheses. The manipulation is designed influence the individual's perception of the temporal proximity of the design task. Therefore, we primed participants with a project description framed in terms of the present or the distant future. Research has shown it is possible to elicit temporal perceptions of the near-term future that are imperceivably from the present (Jones et al., 2017). However, as the timepoint becomes into the distant future, or over ten years, it less likely for individuals perceive the event as they would the present (Gifford, 2011; Soliman et al., 2018). As such, by framing the future condition in distant future, participants were more likely to perceive the design task as significantly different from the present.

"[In 2035,] Sparwood [will be] (is) a caring, neighborly, and sustainable community with pride in its natural environment. A world-class multi-purpose network of trails, parks, and recreational areas [will] support an active, healthy, and highly livable community. A unique and vibrant downtown [will be] (is) the social, cultural, and economic heart of Sparwood. Opportunities to live, work, learn, shop, and play [will be] (are) in close proximity. A diverse economy [will provide] (provides) a range of jobs and services to supplement the mining industry, which [will be] (is) the economic lifeblood. A variety of housing options [will allow] (allows) residents of all income levels and lifestyles to live comfortably in Sparwood [throughout all stages of their lives]."

After the manipulation, participants provided a written description of their conceptual designs for the scenario and also set targets, in years, for the outcome variables of interest: design life, useful life to the community, and maximum acceptable return on investment. By having the three outcome variables of interest relate to different aspects of the wastewater treatment plant's design lifespan the participant's design intentions on projects goals relating to sustainability were measured.

#### Envision framework

After completing the questions on the primary dependent variables, the participants moved on to the experiment section, which dealt with the Envision framework and the secondary dependent variables. Here, the participants provided their targets for ten Envision credits, which pertained to the wastewater facility's sustainable achievement. Participants had to select the level of sustainable achievement for each credit and write a prompt explaining how they would do so. The prompt increased in length if participants set their achievement at higher-levels to simulate the real-world mental effort required for a more sustainable project (Harris et al., 2016). The decision scenario finished with necessary demographic information along with questions on the participant's work history.

As mentioned above, Envision is a sustainable design framework facilitated by the Institute of Sustainable Infrastructure. The framework comprises 60 credits across five different areas: quality of life, leadership, resource allocation, the natural world, climate and resilience, and sustainable design. The achievement levels, for each credit, can range from improved (the lowest level), enhance, superior, conserving, and restorative (the highest level). Depending on a project's achievement for each of these individual applicable credits, and their achievement levels, the Institute of Sustainable Infrastructure will grant an overall project sustainability score. These sustainability certifications include: verified (the lowest level), silver, gold, and platinum (the highest).

After answering the questions to the main dependent variables, they provided their sustainability targets, for the project, via a series of Envision credits. The participants did so by responding to ten credits, presented in random order, from the existing Envision framework. The credits drew from a few different Envision categories, namely: quality of life, leadership, resource allocation, and climate and resilience – see Table 2 for more information. After selecting a sustainability target the participant described, via a written statement, how they would accomplish this sustainability level, without technical specifications. The length of the response increased with each higher sustainability target in order to simulated the cognitive burden of greater achievement.

#### Demographics

The survey ended with participants answering demographic questions.

### Quantification and statistical analysis

Before significance testing the raw data was cleaned, which involved removing any of the participants that failed to pass the attention checks within the survey, and was then tested for normality. Then the data was analyzed using a Welch two sample t-test, due to unequal samples sizes and variance, for each of the three DVs (Useful life to the community, Design life and Longest acceptable return on investment) across experimentally manipulated future and present orientations.

The individual Envision credit scores were analyzed using a multi-level model due to the ten individual credits' repeated measures. The Envision scores were also examined to see if they would predict any of the design metrics.

### Additional resources

#### Preregistration

Before the data collection took place for this study, after a pilot data collection for testing the decision scenario's functioning, the study was preregistered. The preregistration and any other supporting materials can be found on the projects Open Science Foundation page at https://osf.io/z89ve/. To create the preregistration the Aspredicted.org template was used and it listed our hypotheses, intentions for data collection, and data analysis. It should be noted that the original preregistered study design did not include some of the hypotheses from this paper, but all the dependent variables were included in the preregistration. This oversight was left as is in order to not create confusion from a second preregistration.

## Data Availability

• The participant data reported in this study cannot be deposited in a public repository because of a non-disclosure agreement. To request access, contact the Institute of Sustainable Infrastructure and reference this study. • All original code has been deposited at Zenodo and is publicly available as of the date of publication. DOIs are listed in the key resources table. • Any additional information required to reanalyze the data reported in this paper is available from the lead contact upon request.
